# Demographic and Clinical Characteristics Associated With Severity, Clinical Outcomes, and Mortality of COVID-19 Infection in Gabon

**DOI:** 10.1001/jamanetworkopen.2021.24190

**Published:** 2021-09-14

**Authors:** Berthe Amélie Iroungou, Laurette Guignali Mangouka, Berthold Bivigou-Mboumba, Pamela Moussavou-Boundzanga, Judicaël Obame-Nkoghe, Farrel Nzigou Boucka, Augustin Mouinga-Ondeme, Avelin Fobang Aghokeng, Romain Tchoua, Pascal Pineau, Jean Raymond Nzenze

**Affiliations:** 1 Unité Mixte de Recherche Centre International de Recherches Médicales de Franceville et le Service de Santé Militaire, Libreville, Gabon; 2 Service de Médecine Interne, Hôpital d’Instruction des Armées Omar Bongo Ondimba, Libreville, Gabon; 3 Laboratoire de Biologie Moléculaire et Cellulaire, Université des Sciences et Techniques de Masuku, Franceville, Gabon; 4 Unité Écologie des Systèmes Vectoriels, Centre Interdisciplinaire de Recherches Médicales de Franceville, Franceville, Gabon; 5 Agence Gabonaise etudes et d’Observation Spatiale, Libreville, Gabon; 6 Unité Mixite de Recherche, Institut de Recherche pour Developpement 224, Centre National de la Recherche Scientifique 5290, Maladies infectieuses et Vecteurs: Écologie, Génétique, Évolution et Contrôle, Université de Montpellier, Montpellier, France; 7 Service de Réanimation, Hôpital d’Instruction des Armées Omar Bongo Ondimba, Libreville, Gabon; 8 Unité “Organisation Nucléaire et Oncogenèse,” INSERM U993, Institut Pasteur, Paris, France

## Abstract

**Importance:**

Since the emergence of COVID-19 in central China, sub-Saharan African countries, with the exception of South Africa, have been relatively spared during the COVID-19 pandemic. Consequently, few descriptive studies from this region are available.

**Objective:**

To describe the clinical characteristics and outcomes of patients with COVID-19 infection in Gabon, from March to June 2020.

**Design, Setting, and Participants:**

A single-center, cross-sectional study of 837 patients with COVID-19 was conducted from March to June 2020 in the Armed Forces Hospital in Libreville, Gabon.

**Main Outcomes and Measures:**

Demographic and clinical characteristics and imaging findings of hospitalized patients with COVID-19.

**Results:**

Of the 837 patients enrolled, 572 (68.3%) were men, and 264 (31.5%) were women (male to female ratio, 2:1); the median (interquartile range [IQR]) age was 35 (30-45) years (mean [SD] age, 38.0 [12.2] years. The mortality rate associated with COVID-19 was low (1.4%). Of these 837 patients, 524 (62.6%) were categorized as having no symptoms, 282 (33.7%) as having mild symptoms, and 31 (3.7%) as having severe symptoms. Patients with severe symptoms were older (mean [SD] age, 46.1 [14.7] years) than patients with mild symptoms (mean [SD] age, 41.3 [12.5] years) and those with no symptoms (mean [SD] age, 35.7 [11.3] years) (Kruskal-Wallis χ^2^_2_ = 53.5; *P* < .001). History of diabetes was the principal risk factor associated with both severe symptoms in 5 of 31 patients (16.1%) and mild symptoms in 11 of 282 (3.9%) compared with no symptoms in 5 of 524 (0.9%) (Pearson χ^2^_2_ = 30.9; *P* < .001). Patients with severe symptoms and a fatal outcome were older (mean [SD] age, 53.4 [15.1] years) than survivors (mean [SD] age, 41.5 [12.9] years) (*t*_20.83_ = 2.2; *P* = .03).

**Conclusions and Relevance:**

In this single-center, cross-sectional study in Libreville, Gabon, the mortality rate associated with COVID-19 infection from March to June 2020 was low, and patients who died of COVID-19 infection were younger on average than reported elsewhere, possibly reflecting a smaller elderly population in Gabon.

## Key Points

**Question:**

What were the epidemiologic and clinical aspects of patients with COVID-19 infection in the Armed Forces Hospital in Libreville, Gabon, from March to June 2020?

**Findings:**

In this cross-sectional study of 837 patients with COVID-19 in Gabon, 63% had no symptoms. Severity of disease and mortality were associated with advanced age and advanced stage of lung damage.

**Meaning:**

Findings from this observational study provide preliminary data for use in future epidemiologic studies of COVID-19 in Gabon.

## Teaser

This cross-sectional study describes the clinical characteristics and outcomes of patients with COVID-19 infection in Gabon from March to June 2020.

## Introduction

In December 2019, a novel infectious disease, later named COVID-19, was reported in Wuhan, China.^1^ COVID-19 is characterized by a clinical presentation ranging from asymptomatic to severe, the latter of which includes cytokine storm.^2,3^ Older patients or those affected by comorbidities such as cardiovascular diseases, diabetes, or obesity are particularly susceptible to development of severe forms of COVID-19 and are at very high risk for death.^4,5^ After the initial outbreaks in the Chinese province of Hubei, the COVID-19 pandemic has rapidly spread across the world.^6^ Despite concerns about the burden of the COVID-19 pandemic in Africa, African countries, with the exception of South Africa, have reported a relatively low number of cases and a low rate of daily increases in infection.^7^ In Gabon, the first case of COVID-19 was imported from France and reported on March 12, 2020. On March 22, 2020, the Gabonese government established a curfew and partial confinement, including a travel ban on the entire national territory. Three months later, the epidemiologic situation progressed with significant community transmission. By June 15, 2020, 3463 cases of COVID-19 were confirmed, including 23 deaths (a case-fatality rate of 0.66%); by September 2020, 8984 cases of COVID-19 were confirmed, including 55 deaths (a case-fatality rate of 0.61%). This study is focused on the early part of the COVID-19 pandemic in Gabon, when there were initially 2 epidemic foci: Libreville, the national capital on the Atlantic coast, and Franceville in the inner part of the country (eFigure in the Supplement). To date, only 1 retrospective study has been conducted in Gabon^8^; it highlighted cross-reactivity against SARS-CoV-2 nucleocapsid (N) antigen among 23% of samples collected in 2014. To our knowledge, the clinical and epidemiologic characteristics of patients with COVID-19 in Libreville, Gabon, have not yet been described.

## Methods

### Patients

This retrospective cross-sectional study used medical records of patients with COVID-19 who were hospitalized at Hôpital d’Instruction des Armées d’Akanda (HIAA), a military hospital in Libreville, the capital of Gabon, between March 13 and June 15, 2020. Hôpital d’Instruction des Armées d’Akanda is located 16 km north of Libreville. Because of its location and emergency care equipment, HIAA was the only hospital to isolate and treat patients with COVID-19 in Gabon. Surveillance of patients with COVID-19 and their contacts (symptomatic and asymptomatic) was done for at least 14 days. This study was approved by the National Scientific Committee of Gabon and the administration of the HIAA. Written and oral informed consent was obtained from all patients with COVID-19 on admission to the hospital. The study followed the Strengthening the Reporting of Observational Studies in Epidemiology (STROBE) reporting guidelines.

All hospitalized patients with compatible clinical characteristics, confirmed to be infected with SARS-CoV-2 by real-time reverse transcriptase–polymerase chain reaction (RT-PCR) performed using a quantitative RT-PCR system (MA6000; Sansure Biotech Inc) and radiographic findings consistent with COVID-19–related pneumonia on chest computed tomography (CT) within 24 hours of admission, were eligible for inclusion in the study. Clinical outcomes were monitored and recorded daily for follow-up.

According to national guidelines, the PCR testing strategy focused on all people with clinical signs of COVID-19 and contacts of individuals with a positive diagnosis of COVID-19. The surveillance of the epidemiologic situation and the public health measures aiming to combat the pandemic in Gabon were conducted by the National Steering Committee for the Fight Against COVID-19, a structure created by Prime Minister decision 000008/PM on February 25, 2020.^9^

### Clinical Classification

On admission, patients were categorized as (1) severely symptomatic, requiring treatment in the intensive care unit; (2) mildly symptomatic with fever, respiratory symptoms, influenzalike illness (a set of symptoms including fever, shivering, chills, malaise, dry cough, loss of appetite, body aches, and nausea, typically in connection with a sudden onset of illness), or other clinical signs such as anosmia or ageusia; or (3) asymptomatic patients without positive CT findings (or with positive CT findings but no symptoms). Most of the medical files retrieved presented a succinct clinical status. Missing data were mostly from asymptomatic patients who did not benefit, for the most part, from a CT assessment of lung disease because of limited testing capacity.

### Chest CT Score

To standardize the radiologic examinations, a chest CT score algorithm was chosen to determine the severity of pulmonary involvement of COVID-19.^10^ All scans were obtained using a 64-slice CT scanner (Philips Brilliance CT 64; Philips). It was incorporated as a tool to aid the diagnosis of COVID-19–related pneumonia (eMethods in the Supplement). Two radiologists who had 3 and 15 years of experience reviewed all chest CT images independently, and the final decisions reached by consensus were reported.

### Statistical Analysis

Numerical variables were summarized by mean and SD or median and interquartile range (IQR) according to type of distribution (normal or not). They were compared using an unpaired 2-sided *t* test, and for each comparison, the test value (*t*), *df*, and the *P* value are given accordingly. When multiple means were compared according to 1 factor, a 1-way analysis of variance was used. Adjusted *P* values were calculated using the Bonferroni correction method to overcome inflated type I errors due to multiple pairwise comparisons.^11^ In the Bonferroni correction, 3 pairwise tests were used in the denominator, and we considered α = .05/3, that is, .017, as the level of significance for each of the 3 pairwise tests, so that the overall single level of significance for comparison across the 3 levels of symptom severity ended up at .05. Categorical variables were summarized as frequencies and compared using either the Pearson χ^2^ test or the Fisher exact test. For pairwise comparisons of the 3 subgroups of patients using the Pearson χ^2^ test, *df* were equal to 1, whereas comparisons with all the subgroups required *df* to be equal to 2. For the Fisher exact test, the odds ratio (OR) was estimated with a 95% CI. When the Fisher exact test comparisons included null percentages, ORs and 95% CI outputs were described as not applicable. All tests were 2-sided, and the level of significance was set at *P* < .05. For all statistical tests, missing data were described as not available in the original database and were not taken into account during analysis. Analyses were performed using a Prism, version 8.4.2 statistical package (GraphPad) and R software, version 3.6.1 (R Foundation for Statistical Computing).

## Results

According to the National Steering Committee for the Fight Against COVID-19, between March 13 and June 15, 2020, 3463 patients in Gabon had a confirmed diagnosis of COVID-19. Of those, 837 patients were hospitalized at the military hospital (HIAA). The nationality for 253 of the 837 study participants (30.2%) was unknown; of the 584 remaining patients, 556 (95.2%) were Gabonese citizens. Of the 837 patients, 805 (96.2%) were 18 years or older, 572 (68.3%) were men, and 265 (31.7%) were women (Table 1). The median (IQR) age was 35 (30-45) years. The median (IQR) age of deceased patients was 52.5 (45-63.5) years. The number of patients hospitalized who were tested with PCR (nasopharyngeal swab) was 780 vs 57 who were included because of CT changes without PCR testing.

**Table 1.** Clinical Findings Based on Severity of Infection

Characteristic	Patients, No. (%)			*P* value		
	No symptoms	Mild symptoms	Severe symptoms	No symptoms vs mild symptoms	No symptoms vs severe symptoms	Mild symptoms vs severe symptoms
Demographics						
Effect size	524	282	31			
Sex ratio (male to female)	3.5 (407:117)	1.1 (149:133)	1.0 (16:15)	<.001	.001	>.99
Age, median (IQR), y^a^	34 (29-42)	40 (33-49)	46 (34-55)	<.001	<.001	.09
Age, mean (SD), y	35.7 (11.3)	41.3 (12.5)	46.1 (14.7)	<.001	<.001	.09
Clinical symptoms^b^						
ARDS	0	0	31 (100)	NA	<.001	<.001
Influenzalike illness	0	57 (20.2)	0	<.001	NA	.01
Fever	0	36 (12.7)	4 (12.9)	<.001	<.001	>.99
Coughing	0	37 (13.1)	4 (12.9)	<.001	<.001	>.99
Asthenia	0	5 (1.8)	1 (3.2)	<.001	.05	>.99
Digestive signs (nausea, vomiting, diarrhea)	0	5 (1.8)	0	.15	NA	>.99
Anosmia	0	10 (3.5)	0	<.001	NA	.62
Headache	0	48 (17.0)	0	<.001	NA	.007
Dyspnea	0	52 (18.4)	0	<.001	<.001	<.001
Rhinitis	0	2 (0.7)	0	.12	NA	>.99
Clinical parameters at admission^a^						
Body temperature, median (IQR), °C	36.0 (36.0-36.0)	37.0 (36.0-37.6)	36.5 (36.0-37.2)	<.001	.13	.75
Systolic blood pressure, median (IQR), mm Hg	120.5 (115.5-130.8)	124.0 (110.0-125.5)	115.0 (107.5-122.5)	.90	.40	.50
Diastolic blood pressure, median (IQR), mm Hg	80.0 (72.8-90.0)	80.0 (70.0-85.8)	75.0 (67.5-82.5)	.07	.60	.70
Heart rate, median (IQR), /min	78.0 (73.0-91.0)	80.0 (71.0-88.0)	79.5 (63.0-85.8)	.85	.99	.81
Oxygen saturation, median (IQR), %	98.0 (98.0-99.0)	98.0 (97.0-99.0)	93.0 (91.0-94.0)	.19	<.001	
Antecedent^b^						
Tobacco consumption	3 (0.5)	4 (1.4)	1 (3.2)	>.99	.37	.40
Alcohol consumption	10 (3.5)	19 (6.7)	1 (3.2)	.18	>.99	.70
Asthma	4 (0.8)	5 (1.8)	1 (3.2)	.29	.25	.46
Obesity	0	5 (1.8)	0	.005	NA	>.99
Diabetes	5 (2.0)	11 (3.9)	5 (16.1)	.006	<.001	.013
Hypertension	13 (2.5)	23 (8.2)	6 (19.4)	<.001	<.001	.05
Pregnancy	0	1 (0.004)	0	.05	NA	>.99
Tumor diseases	0	1 (0.004)	0	.05	NA	>.99
HIV/AIDS	0	1 (0.4)	2 (6.4)	.35	.003	.02
Positive thoracic scan (%)^b^	0	0	0 (19.3)	NA	<.001	NA
Isolation motives (%)^b^						
Positive case	473 (90.3)	250 (88.5)	28 (91.0)	.46	>.99	>.99
Contact	51 (9.7)	32 (11.5)	3 (9.0)	.46	>.99	>.99
Place of infection (%)^b^						
Workplace	445 (84.9)	217 (76.6)	19 (61.2)	.006	.001	.07
Home	79 (15.1)	47 (16.8)	6 (19.3)	.54	.45	.80
Transports	0	18 (6.5)	6 (19.3)	<.001	<.001	.02

Abbreviations: ARDS, acute respiratory distress syndrome; IQR, interquartile range; NA, not applicable.

^a^ Numerical variable.

^b^ Categorical variable.

### Overview of the Different Forms of COVID-19 Affecting Gabonese Patients

#### Clinical Classification

We stratified the 837 patients with COVID-19 into 3 categories: 31 (3.7%) with severe symptoms, 282 (33.7%) with mild symptoms, and 524 (62.6%) with no symptoms. Sex ratios (male to female) in patients with severe symptoms (16:15) and mild symptoms (149:133) were balanced, but more men than women (407:117) were represented among patients with no symptoms.

Clinical variables describing the 3 categories of patients are summarized in Table 1. Patients with severe COVID-19 symptoms who were admitted to the intensive care unit and affected by acute respiratory distress syndrome were older (mean [SD] age, 46.1 [14.7] years) than those with mild symptoms (mean [SD] age, 41.3 [12.5] years) and those with no symptoms (mean [SD] age, 35.7 [11.3] years) (analysis of variance *F*_2_ = 27.6; *P* < .001) (Figure).

**Figure.**
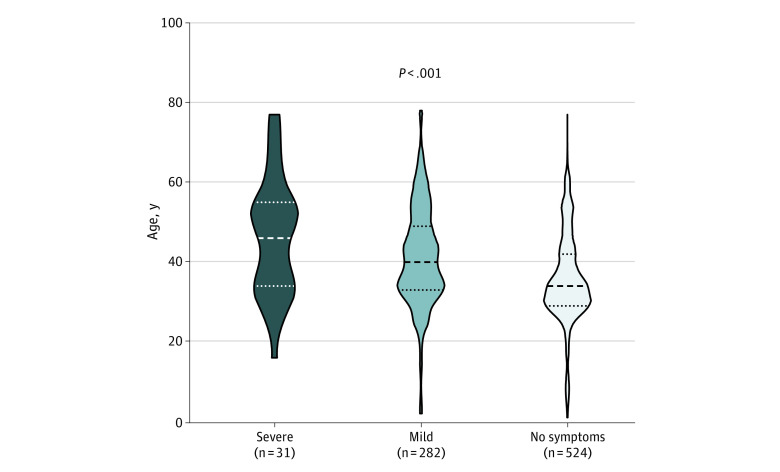
Age Differences Among Patients With Severe Symptoms, Mild Symptoms, and No Symptoms of COVID-19 Patients with severe symptoms of COVID-19 admitted to the intensive care unit and affected by acute respiratory distress syndrome were significantly older than those with mild symptoms. The horizontal lines in the data markers represent the median (dashed line) and the first and third quartiles (dotted lines) for each group of patients.

In terms of clinical presentation, the most frequently observed clinical signs were as follows: influenzalike illness, 57 patients (20.2%) with mild symptoms and no patients with severe symptoms or no symptoms; fever, 4 patients (12.9%) with severe symptoms, 36 (12.7%) with mild symptoms, and none with no symptoms; and coughing, 4 patients (12.9%) with severe symptoms, 37 (13.1%) with mild symptoms, and none with no symptoms. Asthenia, digestive signs (nausea, vomiting, and diarrhea), anosmia, headaches, dyspnea, and rhinitis were each observed in fewer than 5% of patients regardless of severity of disease (Table 1).

On comparison of these occurrences in the 3 categories of patients, influenzalike illness was significantly more frequent in patients with mild symptoms (57 patients [20.2%]) than in patients with severe symptoms (0%) and patients with no symptoms (0%) (Pearson χ^2^_2_ = 120.4; *P* < .001). Fever among patients with severe symptoms (4 patients [12.9%]) and those with mild symptoms (36 [12.7%]) was similar (Pearson χ^2^_1_ = 0; *P* > .99). However, occurrences of both influenzalike illness and fever were more frequent than in patients with no symptoms (0%) (Pearson χ^2^_2_ = 70.3; *P* < .001). Similarly, there was no difference in coughing between patients with severe symptoms (12.9%) and those with mild symptoms (13.1%) (Pearson χ^2^_1_ = 0; *P* > .99). The other clinical symptoms—including digestive signs, anosmia, headache, dyspnea, and rhinitis—were mainly reported in patients with mild symptoms (Table 1). However, asthenia was only observed in 1 of 31 patients (3.2%) with severe symptoms and 5 of 282 (1.8%) with mild symptoms. Anosmia was reported in 10 patients with mild symptoms, but the difference was not significant compared with both patients with severe symptoms and those with no symptoms considered jointly (OR, 0.0; 95% CI, 0-3.0; *P* = .62).

In terms of continuous variables, including blood pressure and heart rate, no significant difference was observed between patients with severe and mild symptoms (Table 1). However, mean (SD) values for body temperature were significantly higher in patients with mild symptoms compared with patients with no symptoms (36.9 °C [0.7 °C] vs 36.2 °C [0.4 °C]; *U* = 2833; *P* = .001), although this difference was 0.7 °C. The differences that we observed in pairwise comparisons of body temperature in patients with severe symptoms were not significant. We found that the mean value of oxygen saturation was significantly lower in patients with severe symptoms (93%; IQR, 91%-94%) than in patients with no symptoms (98%; IQR, 97%-99%) and those with mild symptoms (98%; IQR, 98%-99%) considered together (1-way analysis of variance *F*_2_ = 36.7; *P* < .001).

#### Comorbidities

A history of diabetes was significantly more frequent in patients with severe symptoms (16.1%) than in patients with mild symptoms (3.9%) and those with no symptoms (0.9%) when considered together (Pearson χ^2^_2_ = 30.9; *P* < .001) or separately (Table 1). Arterial hypertension was significantly more frequent in patients with severe symptoms (6 patients [19.4%]) than in both patients with mild symptoms (23 [8.2%]) and those with no symptoms (13 [2.5%]) considered jointly (Pearson χ^2^_2_ = 26.3; *P* = .001) or separately (Table 1). However, the difference between patients with severe symptoms and those with mild symptoms was nonsignificant (Pearson χ^2^_1_ = 2.9; *P* = .05). Among patients with HIV or AIDS, the difference between those with mild symptoms and those with no symptoms was also nonsignificant. However, we observed that patients with HIV or AIDS were more, likely to have severe symptoms than mild symptoms (Fisher exact test OR, .05; 95% CI, 0.0008-1.4; *P* = .02).

#### Characterization of Severe COVID-19 Symptoms With Fatal Outcome

Among patients with severe symptoms, those with a fatal outcome were older (12 patients [38.7%]; mean [SD] age, 53.4 [15.1] years) than survivors (19 patients [61.3%]; mean [SD] age, 41.5 [12.9] years) (*t*_20.83_ = 2.2, *P* = .03). No comorbidities were significantly associated with a fatal outcome.

#### COVID-19 and Age

Older age seemed to be associated with a higher mortality rate in this study. However, the proportion of patients older than 65 years in the present series was very low (17 of 832 patients [2.0%]). To identify relevant biological parameters capable of anticipating deterioration of health status, we stratified patients with milder forms of the disease according to the median age (41 years) and used this cutoff to stratify patients with no symptoms.

#### Chest CT Assessment and Images

Lung abnormalities were recorded as subpleural (mainly involving the peripheral third of the lung), random (without predilection for subpleural or central regions), or diffuse (involvement of all lung segments). Specifically, 448 of 837(53.5%) had missing CT scan data.

The most common patterns on chest CT were ground-glass opacity and bilateral patchy shadowing. We identified 4 stages of lesions on the CT scan (eMethods in the Supplement); 230 of 389 patients (59.13%) had a CT score of stage II (6%-25% of pulmonary abnormalities corresponding to parenchymal and subpleural solid nodules). We noticed that advanced thoracic CT scores for stages III and IV occurred more frequently in patients who died (6 of 9 patients [66.7%]) than among those who survived (no patients) (OR, 0.0; 95% CI, 0.0-0.2; *P* < .001). We observed that there was no significant difference between men and women for CT scores for stages I to III (Table 2). However, a male predominance was observed for CT score of stage IV: 13 of 193 men (6.7%) vs 2 of 196 women (1.0%) (OR, 6.9; 95% CI, 1.5-64.6; *P* = .003) (Table 2).

**Table 2.** Lung Abnormalities Detected in Patients According to Sex and Percentage of Lesions Revealed by Thoracic Computed Tomography^a^

Lesions, %	No. (%)		
	All patients (N = 389)	Men (n = 193)	Women (n = 196)
0-5	91 (23.4)	44 (48.4)	47 (51.6)
6-25	230 (59.1)	110 (47.8)	120 (52.2)
26-50	53 (13.6)	26 (49.1)	27 (50.9)
>50	15 (3.9)	13 (86.7)	2 (13.3)

^a^ Lesions detected by thoracic computed tomography were reported as percentages. Percentages for data by sex were calculated using the total number of patients in that row as the denominator.

## Discussion

To our knowledge, this preliminary study, which was conducted during the early part of the COVID-19 pandemic, is among the first to report clinical characteristics and outcomes of hospitalized Gabonese patients with COVID-19. Like other countries in Africa, Gabon has reported a low number of SARS-CoV-2 cases since detection of the first case on March 12, 2020. On March 22, 2020, the Gabonese government mandated mask wearing in public places, curfews, and partial confinement, including a travel ban, on the entire country. These actions during the early part of the pandemic may have helped to mitigate the spread of the virus. Moreover, as suggested by Njenga et al,^12^ other factors, such as temperature, population density, prior exposure to other coronaviruses, and younger population, may have played a role.^12^

A few prior publications have described the clinical findings of COVID-19 in individuals living in sub-Saharan Africa.^13^ In the present study, most patients (62.6%) had no symptoms and, among the 37.4% of patients with symptoms, 33.7% had mild symptoms and 3.7% had severe symptoms. None of the patients observed in this study were children, although cases in pediatric patients have been reported in the Democratic Republic of the Congo and Nigeria.^13^ In this study, we observed that older age and comorbidities, specifically diabetes and arterial hypertension, were associated with more severe forms of COVID-19 and were thus considered to be risk factors for severe symptoms. This appears to be consistent with findings from other studies outside of sub-Saharan Africa.^5,14,15^ Moreover, a greater percentage of men was observed among patients with severe or mild symptoms of COVID-19 in Gabon (Table 2), which is similar to the finding in Congolese cohorts.^16^ Otuonye et al^17^ made the same observation in a study conducted in Nigeria.^17^ This has also been described for SARS-CoV and Middle East respiratory syndrome coronavirus (MERS-CoV) and may be associated with the protective role of the X chromosome and sex hormones, which are known to play an essential role in innate and adaptive immunity.^18^

The relative mortality rate for patients with COVID-19 in Gabon is estimated to be low (0.6%) according to national statistics, compared with the corresponding rate in South Africa and in France (both 2.6%).^19^ The lower mortality rate observed in our study may be a result of its conduction within a tertiary referral hospital that admits patients with severe COVID-19 symptoms. We examined parameters associated with fatal outcomes in 12 of 31 patients (38.7%) admitted to the intensive care unit. The only parameter significantly associated with death was older age. The median age of deceased Gabonese patients was 52.5 years; however, it is much lower than the median ages observed in China (68.5 years), Iran (65.4 years), and Italy (79.6 years).^20,21,22^ With regard to comorbidities, diabetes and arterial hypertension were more prevalent in individuals who died than in those who recovered, although the differences were not statistically significant. Numerous other studies have reported a positive association between mortality and diabetes or arterial hypertension.^5,14,23^ In terms of chronic infectious diseases, HIV and AIDS were the only chronic infectious diseases observed in our study. However, because of the limited sample size, we could not assess the association between HIV and risk of death associated with COVID-19. Nevertheless, Boulle et al,^24^ in their study population in South Africa, found that comorbidities such as HIV and tuberculosis were more likely to increase the risk of COVID-19 mortality.

In terms of chest CT data, ratios of positive imaging findings were, as expected, higher in patients with severe symptoms of COVID-19. Meftahi et al^25^ have shown that in patients with COVID-19 pneumonia, cytokine storm results in the appearance of acute interstitial lung lesions, alteration of the pulmonary parenchyma, and pulmonary edema with alveolar cell exudates. The pulmonary abnormalities observed in patients from Libreville are in agreement with findings of previous studies showing that the most common lung lesions observed are a ground-glass appearance, the “crazy-paving” pattern, and focal consolidation related to internalization of the virus in the pneumocytes.^26^ The crazy-paving pattern describes the presence of multiple ground glass opacities mixed with interlobular and intralobular thickening.

The favorable demographic configuration in sub-Saharan Africa with regard to COVID-19 pathophysiology has been discussed by other authors.^27,28,29^ In our series, the potential deleterious impact of local endemic diseases, such as malaria, chronic hepatitis, tuberculosis, and AIDS, did not clearly appear.^30^

### Limitations

This study had several limitations, including the small number of patients in the intensive care unit and missing data on outcomes of interest. Additional patient data from other cities around the country should be added to gain a more comprehensive understanding of the clinical characteristics of the disease in Gabon. Moreover, it is a single-center study that is not necessarily representative of the whole region.

## Conclusions

We believe this cross-sectional study, carried out in Gabon, presents the most extensive description, to date, on the clinical and demographic characteristics of patients with COVID-19. To our knowledge, it is the first in which a low proportion of severe cases (3.7% in the present series) and a lower mortality rate (1.4%) were observed in Gabon. These findings are consistent with the low proportion of elderly individuals (>65 years) in the present series (2.0%) as in the Gabonese population in general (5.1%).

## Notes

Supplement. **eMethods.** Chest Computed Tomography Score **eFigure.** Epidemic Foci in Libreville and Franceville
