# Supplementary material for: Demographic and Clinical Characteristics Associated With Severity, Clinical Outcomes, and Mortality of COVID-19 Infection in Gabon
Source: JAMA Netw Open. 2021 Sep 14;4(9):e2124190. doi: 10.1001/jamanetworkopen.2021.24190 (PMC8441589; doi:10.1001/jamanetworkopen.2021.24190)
Supplement: Supplement. — eMethods. Chest Computed Tomography Score eFigure. Epidemic Foci in Libreville and Franceville [file jamanetwopen-e2124190-s001.pdf]

# Supplementary Online Content

Iroungou BA, Mangouka LG, Bivigou-Mboumba B, et al. Demographic and clinical characteristics associated with severity, clinical outcomes, and mortality of COVID-19 infection in Gabon. *JAMA Netw Open*. 2021;4(9):e2124190. doi:10.1001/jamanetworkopen.2021.24190

**eMethods.** Chest Computed Tomography Score

**eFigure.** Epidemic Foci in Libreville and Franceville

This supplementary material has been provided by the authors to give readers additional information about their work.

© 2021 Iroungou BA et al. *JAMA Network Open*.

**eMethods. Chest Computed Tomography Score**

We incorporated the chest CT-scan as a tool to aid in the diagnosis of "COVID-19 related pneumonia". A semi-quantitative scoring system was used to estimate lung involvement from all abnormalities. For each patient, the chest CT scan was evaluated for the following characteristics based on the Fleischner Society Nomenclature recommendations 14: ground glass opacity (GGO), consolidation, nodule, reticulation, interlobular septal thickening, crazy-paving pattern, linear opacities, subpleural curvilinear line, bronchial wall thickening, lymph node enlargement, pleural effusion, and pericardial effusion. The "spider web sign" was defined as a triangular or angular GGO in the subpleural lung with the internal interlobular septa thickened like a net; the adjacent pleura was pulled and formed a spider web–like shape in the corner. To quantify the extent of lesions, a thin-section CT score was assigned based on all abnormal areas involved. Each lobe was assigned a score that was based on the following: score 0, 0% involvement; score 1, less than 5% involvement; score 2, 5% to 25% involvement; score 3, 26% to 49% involvement; score 4, 50% to 75% involvement; and score 5, greater than 75% involvement. Then, each score allowing us to classify lung lesions into 4 stages including: <5% (CT stage I) of lung damage, between 5 to 25% (CT stage II), >25-50% (CT stage III) and >50% (CT stage IV).

© 2021 Iroungou BA et al. *JAMA Network Open*.

## eFigure. Epidemic Foci in Libreville and Franceville

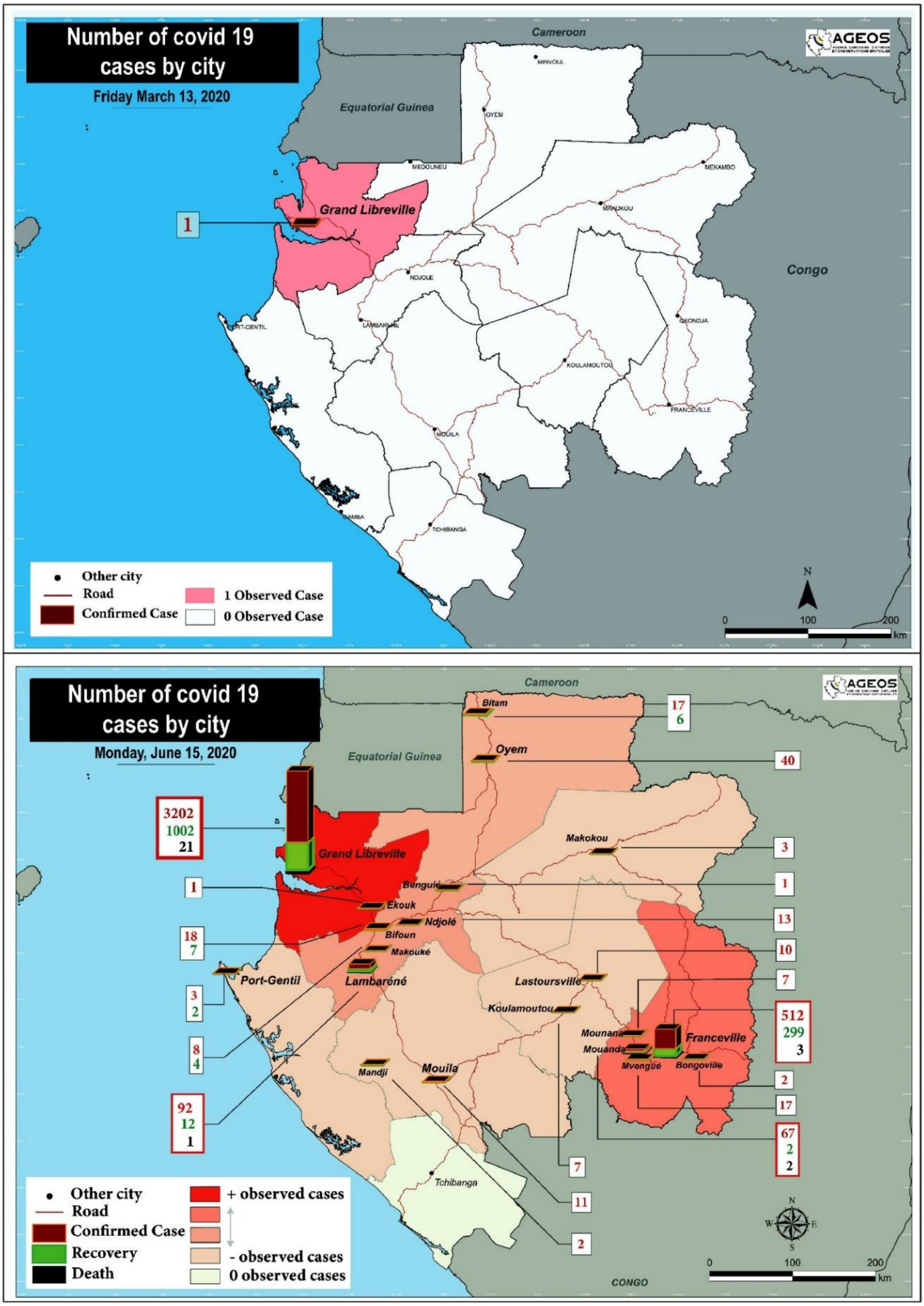


© 2021 Iroungou BA et al. *JAMA Network Open*.
